# Food Craving and Its Relationship with Restriction and Liking in Japanese Females

**DOI:** 10.3390/foods3020208

**Published:** 2014-04-16

**Authors:** Sakura Komatsu, Kenjiro Aoyama

**Affiliations:** 1 Organization for Advanced Research and Education, Faculty of Psychology, Doshisha University, 1-3 Tatara Miyakodani, Kyotanabe, 610-0394, Japan; 2 Faculty of Psychology, Doshisha University, 1-3 Tatara Miyakodani, Kyotanabe, 610-0394, Japan; E-Mail: kaoyama@mail.doshisha.ac.jp

**Keywords:** food craving, restriction, liking, rice craving, Japanese

## Abstract

Craved foods are thought to be those that are well liked but restricted. However, this claim has not been demonstrated empirically. Japanese female undergraduate students (*n* = 144) completed a questionnaire measuring their craving for, degree of liking, and frequency of restricting their eating of 47 widely consumed foods. The food with the highest mean craving score was rice. We plotted the craving scores as a function of restriction and liking scores for the 47 foods. The students’ craving scores were strongly correlated with their restriction scores and liking scores. Thus, craved foods are those that are restricted and liked. However, in both scatter plots, rice was an outlier. While it was the most craved food, neither the restriction nor liking score of rice was very high. These findings were consistent with the view that craved foods are generally liked, yet restricted, implying the generation of food related conflicts. Interestingly, the mechanism of craving rice, the main staple in Japan, may differ from other foods.

## 1. Introduction

When we sometimes feel an irresistible or intense desire to consume a particular food, this phenomenon is defined as a food craving [1,2]. It is apparent that people have a tendency to crave certain foods [2,3]. As for the foods that people crave and demographic factors associated with the cravings, studies have found that chocolate is a commonly craved food, especially by women in Western cultures [2,4,5,6]. In contrast, Komatsu [7] reported that Japanese women most frequently crave rice. These divergent findings indicate that the characteristics of foods associated with food cravings are not obvious or easily distinguished. Although Mann and Ward [8] suggested that craved foods are liked but restricted, this claim has not been demonstrated empirically. Therefore, the first aim of the current study was to examine whether craved foods are those that are liked but restricted.

The results of several studies suggest that individual differences should be taken into account in research on food cravings, since large individual differences have been reported in studies of restrained eating of craved foods [9,10]. However, the findings of many of the studies are not definitive. Although some studies found that restrained eaters more frequently experienced food cravings than unrestrained eaters [11,12,13,14,15], others reported no relationship between restrained eating and food cravings [7,16,17,18,19,20]. To clarify the mixed results of the existing research, the second aim of this study was to examine whether individual differences in restrained eating are related to individual differences in food cravings. We expected that the participants with high restriction scores would tend to have high craving scores.

The inconsistency in the research findings may be attributed to the different assessment methods used in the studies. For example, in a study that found a strong relationship between restrained eating and self-reported food cravings [12] asked women, “How often do you experience strong urges to eat particular types of food?” The researchers measured restrained eating using the Dutch Eating Behaviour Questionnaire [21] and the Three-Factor Eating Questionnaire [22]. In contrast, a study that found no relationship between restrained eating and food cravings [17] examined the frequency of a craving for chocolate and current dieting state. These studies and others [7,11,12,13,14,15,16,17,18,19,20] were similar in terms of defining restrained eating (i.e., restricting the eating of the amount and types of food one eats to reduce energy intake). However, there were differences in the target of the measures among the studies. Although earlier studies [11,12,13,14,15] assessed general food cravings, later studies [7,16,17,18,19,20] assessed cravings for specific foods. The findings indicated a relationship between general food cravings and restrained eating, while there was no relationship between cravings for specific foods and restrained eating. Thus, it was inferred that there might be a relationship between individual differences in cravings for specific foods and individual differences in the restriction of the specific foods.

A basic premise of craving studies is that people crave foods that they like. Rozin *et al*. [4] showed that chocolate was the most craved food among females, and a very well liked one in America. Similarly, both American and Spanish females liked chocolate-flavored foods and chose chocolate as their main craving [6]. However, it has not been directly examined whether the frequency or intensity of food cravings correlate with the degree of liking. Therefore, the current study also investigated whether individual differences in liking certain foods are associated with individual differences in cravings for the certain foods. We expected that the participants with high liking scores would tend to have high craving scores for certain foods.

## 2. Method

### 2.1. Participants & Procedure

The participants in this study were 144 female undergraduate students with a mean age of 20.15 years (standard deviation (SD) = 1.92 years). The questionnaires for the study were distributed to the students during class time and they were asked to return them within one week. A consent form explained the purpose of the research. Participants were informed that they could refuse to answer the questions, stop answering whenever they wished, and that they did not have to write their names on the questionnaires. Students who agreed to participate in the study signed the consent form and answered the questionnaire. After completing the questionnaire, participants were compensated with course credits. The Institutional Review Board of Doshisha University approved the study.

### 2.2. Measures

The questionnaire assessed each participant’s frequency of craving, the degree of liking, and the frequency of restriction for 47 food items (Table 1). At the top of the questionnaire, the following explanation appeared: “A food craving is defined as an intense desire to consume a particular food (or food type). This desire is so strong that the person feels difficulty resisting.” This question followed, “Over the past month, how often have you experienced a craving for each of the 47 foods listed below?” The response options for the question used a five-point Likert-type scale, ranging from 0 (never) to 4 (always/almost every day). Following the questions about craving, participants were asked the question “How much do you like the foods listed below?” The response options for the question were on a six-point Likert-type scale ranging from 0 (extremely dislike) to 5 (like extremely well). Finally, participants were asked to answer the question “Over the past month, how often have you refrained from eating the foods listed below, reduced eating frequency, or reduced the amount of consumption to less than you wanted to eat?” The response options for the question were on a five-point Likert-type scale ranging from 0 (never) to 4 (always).

## 3. Results

Table 1 shows the mean craving scores and standard deviations for the 47 foods with the means presented in descending order. The results were consistent with those reported by Komatsu [7]. Among our participants, the most frequently craved food was rice. Sweets, such as chocolate and cake, and Chinese noodles followed. Table 2 shows the five items with the highest mean craving scores, as well as the means and standard deviations of restriction and liking scores. We focused on the top food items, based on their mean craving scores, because the craving scores for most the food items were low. Thus, there was the possibility of a floor effect.

**Table 1:** Means (standard deviations, SD) of craving scores.

Foods	Means (SD)	Foods	Means (SD)	Foods	Means (SD)
Rice	2.35 (1.44)	Fried chicken	1.37 (1.28)	Oden	0.94 (1.01)
Chocolate	2.22 (1.25)	Okonomiyaki	1.35 (1.16)	Toro	0.88 (1.21)
Cake	2.10 (1.22)	Curry rice	1.34 (1.06)	Yakiimo	0.85 (1.05)
Ice cream	1.98 (1.25)	Udon	1.34 (1.17)	Ochazuke	0.83 (0.99)
Chinese noodles	1.78 (1.19)	Takoyaki	1.28 (1.15)	Soba	0.81 (1.00)
Pasta	1.74 (1.22)	Donuts	1.23 (1.00)	Anko	0.80 (1.06)
Yakiniku	1.65 (1.30)	Tonkatsu	1.19 (1.04)	Manju	0.79 (1.04)
Onigiri	1.54 (1.28)	Pizza	1.19 (1.11)	Nankotsu	0.79 (1.09)
Nigirizushi	1.48 (1.35)	Candy	1.16 (1.21)	Makizushi	0.76 (1.02)
Fried potato	1.47 (1.15)	Cyuka-man	1.15 (1.09)	Bacon	0.74 (0.97)
Pudding	1.46 (1.27)	Biscuits	1.09 (1.13)	Tempura	0.74 (0.92)
Cookies	1.44 (1.21)	Sandwich	1.05 (1.08)	Hot dog	0.72 (0.98)
Cream puff	1.44 (1.22)	Sausage	1.02 (1.01)	Inarizushi	0.66 (0.96)
Donburi	1.44 (1.19)	Dango	1.01 (1.09)	Yokan	0.58 (0.89)
Hamburger	1.42 (1.13)	Kimuchi	0.99 (1.16)	Nuts	0.54 (0.88)
Potato chips	1.38 (1.13)	Steak	0.99 (1.14)		

For descriptions of Japanese foods, see Komatsu [7].

**Table 2:** Means (SD) of the five items with the highest mean craving scores.

Foods	Restriction Means (SD)	Liking Means (SD)
Rice	1.00 (1.21)	3.83 (1.24)
Chocolate	1.68 (1.33)	3.98 (1.16)
Cake	1.62 (1.27)	4.16 (1.08)
Ice cream	1.29 (1.19)	3.94 (1.11)
Chinese noodles	1.22 (1.24)	3.69 (1.16)

**Figure 1:**
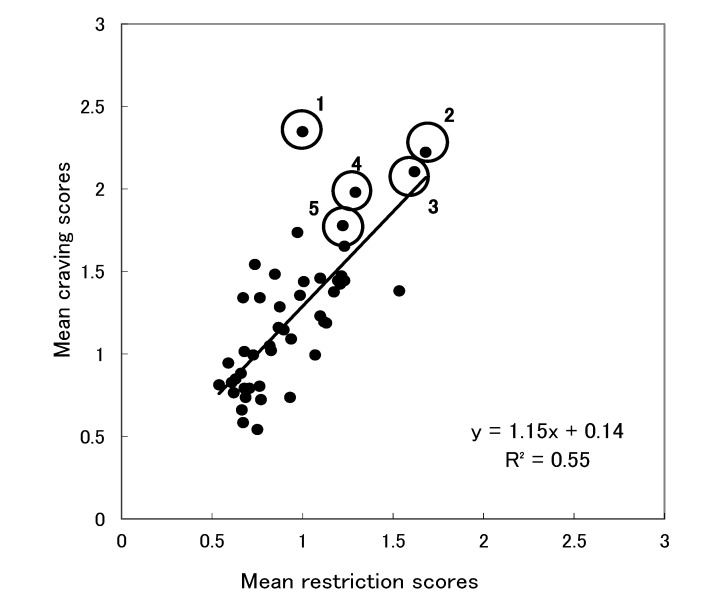
Scatter plot of the mean restriction scores and the mean craving scores.

Note: 1, Rice; 2, Chocolate; 3, Cake; 4, Ice cream; 5, Chinese noodles.

To examine the relationship between the frequency of craving for the 47 foods and the frequency of restriction of the foods, we plotted craving scores and restriction scores for all the foods (Figure 1). Each point represents one food item. The vertical axis indicates mean craving scores, and the horizontal axis indicates mean restriction scores. The results revealed that the craving scores had a strong positive correlation with the restriction scores, *r* (45) = 0.74, *p* < 0.01. The five foods with the highest craving scores, except for rice, tended to be those most frequently restricted. Rice seemed to depart from this trend. That is, although rice had the highest craving score, the restriction score was moderate. To confirm this visual inspection statistically, we conducted the analysis below. We calculated the distance from the observed value to the regression line (*y* = 1.15*x* + 0.14) for the five foods with the highest craving scores for each participant. The distance was calculated using the length of the perpendicular line drawn from each of the observed values to the regression line. Table 3 shows the means of the distances from the observed values to the regression line for the five items with the highest mean craving scores. To examine whether the differences in the distances were different depending on the food, we performed a one-way ANOVA of the distances. The results showed a significant effect of food, *F* (4, 715) = 4.31, *p* < 0.01. The follow-up comparisons showed that rice had a significantly longer distance from the regression line than the other four foods, *t* (715) > 2.92, *p* < 0.01. The other four foods did not differ from each other, *p* > 0.52.

**Table 3:** Means (SD) of the distances between the observed values and the regression line for the five items with the highest mean craving scores in the scatter plot.

	Rice Means (SD)	Chocolate Means (SD)	Cake Means (SD)	Ice cream Means (SD)	Chinese noodles Means (SD)
**Restriction**	1.13^a^ (0.85)	0.86^b^ (0.71)	0.87^b^ (0.70)	0.84^b^ (0.70)	0.82^b^ (0.73)
**Liking**	0.87^a^ (0.50)	0.63^b^ (0.46)	0.65^b^ (0.46)	0.66^b^ (0.50)	0.59^b^ (0.47)

Means with different superscripts are significantly different to each other (*p* < 0.01).

**Figure 2:**
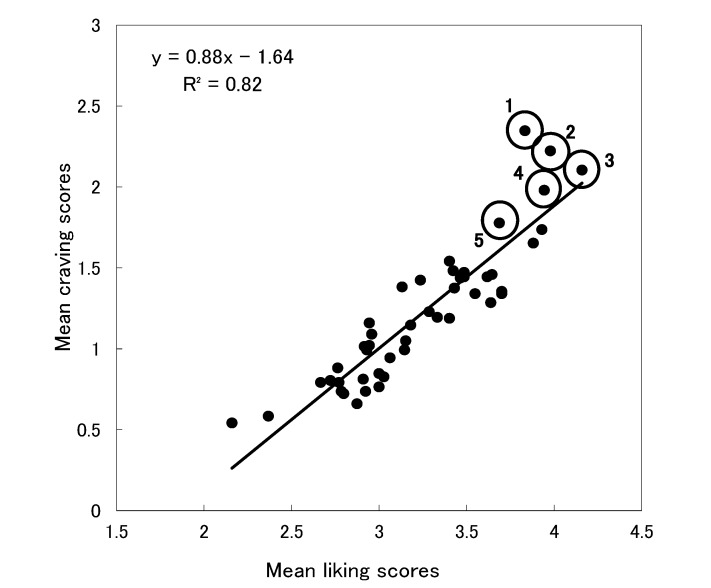
Scatter plot of the mean liking scores and the mean craving scores.

Note: 1, Rice; 2, Chocolate; 3, Cake; 4, Ice cream; 5, Chinese noodles.

Figure 2 plots the mean scores of liking on the horizontal axis. The plot revealed a strong positive correlation between the liking and craving scores, *r* (45) = 0.91, *p* < 0.01. The five foods with the highest craving scores tended to be those most liked, except for rice. Although rice was not the most liked food, it had the highest craving score. As done in the case of plotting the mean restriction and mean craving scores, we performed a one-way ANOVA on the distances between the observed values and the regression line for the five foods with the highest craving scores (Table 3). The results demonstrated a significant effect of food, *F* (4, 715) = 5.07, *p* < 0.01. Follow-up comparisons showed that rice had a significantly longer distance to the regression line than the other four foods, *t* (715) > 2.94, *p* < 0.01 indicating that the four foods did not differ from each other, *p* > 0.23.

To examine whether individual differences in restrained eating are related to individual differences in food cravings, we conducted a correlation analysis of the craving and restriction scores for each of the five items having the highest craving scores (Table 4). There was a significant positive correlation between the craving and restriction scores, except for rice. These results suggest that persons with high restriction scores for chocolate tended to have high craving scores for chocolate. Similar trends were observed between the restriction and craving scores for cake, ice cream, and Chinese noodles. However, this correlation was not observed between the restriction and the craving scores for rice. In other words, individual differences in the restriction scores for rice were not related to the individual differences in the craving scores for rice. Similarly, to examine whether individual differences in liking certain foods are related to individual differences in food cravings, we conducted a correlation analysis of the craving and liking scores. Craving scores were significantly correlated with liking scores for the top five foods craved. These results suggest that persons with high liking scores for rice tended to have high craving scores for rice. Similar trends were observed between the liking and craving scores for chocolate, cake, ice cream, and Chinese noodles.

**Table 4:** Correlations between craving and restriction and between craving and liking for the five items with the highest mean craving scores.

Foods	Restriction	Liking
Rice	0.11	0.62 **
Chocolate	0.27 **	0.65 **
Cake	0.20 *	0.55 **
Ice cream	0.23 **	0.54 **
Chinese noodles	0.21 *	0.60 **

** *p* < 0.01, * *p* < 0.05.

## 4. Discussion

The first aim of this study was to examine whether craved foods were those that were liked but restricted. Our findings showed that craved foods were, in general, frequently restricted (see Figure 1). Although rice was the most frequently craved food, it was not the most frequently restricted one. Similarly, craved foods in general, were extremely well liked (see Figure 2). Although the liking score for rice was not very high, the frequency of craving it was the highest. Our research demonstrated that craved foods consist of restricted and liked foods. This result is similar to the previous findings that dieters felt more cravings for foods that they were restricted from eating [14]. Rogers and Smit [23] also noted that attempts to restrict certain foods were associated with cravings for the foods. However, rice departed from this trend, indicating that restriction and liking alone cannot account for rice as a craved food among Japanese individuals. Therefore, other factors may have influenced cravings for rice.

The second aim of this study was to examine whether there was a relationship between individual differences in the restrained eating of specific foods and individual differences in their cravings for specific foods. In the highest four out of five food craving scores, the results revealed a significant positive correlation between craving scores and restriction scores. Previous studies found no relationship between restrained eating and food cravings for specific foods [7,16,17,18,19,20]. Therefore, the restriction of specific foods (excluding lower energy intake and general food restriction), may influence cravings for specific foods. Polivy *et al*. [24] examined whether deprivation of a food had an effect on intake and craving for that food. They showed that being deprived of chocolate causes a craving for chocolate, as measured by the amount of chocolate consumed and eating latency. However, there was no effect of deprivation on self-reports of cravings. It appears that the results of studies measuring deprivation may be dependent on the instrument used to measure the cravings.

The relationship between the restriction of rice and cravings for rice differed from other foods. The restriction of rice did not correlate with rice craving. In the current study, restrained eating was defined as restricting eating or reducing the amount of consumption by one’s own will, that is, “voluntary restriction”. However, food restriction does not have to be voluntary. For example, people may not eat specific foods, not because they voluntarily refrain from doing so, but because they cannot obtain those foods. Although rice craving did not correlate with “voluntary restriction”, it may correlate with “involuntary restriction.” This view is based on the observation that Japanese individuals do not frequently restrain themselves from eating rice voluntarily. Although rice is a staple food that Japanese people eat at almost every meal, sometimes they cannot eat rice because of the time needed to prepare it. Therefore, a future study should examine whether there is a relationship between a craving for rice and the involuntary restriction of rice.

For the top five foods, craving scores positively correlated with liking scores, indicating an association between the two variables. In addition, the correlation between the craving scores and the liking scores was stronger than the correlation between the craving scores and the restriction scores.

## 5. Conclusions

In conclusion, the present study demonstrated that craved foods were liked and restricted. The craving for foods, excluding rice, correlated with restrained eating of the foods. However, factors other than restriction are likely to contribute to craving. For example, exposure to food cues increased craving for the cued food [9,10]. Situational circumstances also have an effect on the occurrence of food cravings. Cravings for savory foods (e.g., crisps, pizza, and toast) were more frequently reported at home [5], which may be related to people’s usual routine of eating those foods as a meal at home. Therefore, it is not surprising that Japanese people may crave rice at mealtime because they ordinarily eat rice as a staple at almost every meal.

The present study has several limitations. We did not collect data on the participants’ characteristics, such as their dietary habits and body mass index (BMI). In addition, this was a correlation study that can only reveal associations between variables, not causal relationships. Future experimental studies are needed to examine causality. For instance, controlling the restriction of an unfamiliar, highly palatable food after developing a craving for it could help to determine the causal processes involved in food cravings.
